# GRIK1 genotype and effect of topiramate for alcohol use: a systematic review

**DOI:** 10.1186/s40780-025-00449-y

**Published:** 2025-05-20

**Authors:** Kazumasa Kotake, Satoru Matsunuma

**Affiliations:** 1 Department of Pharmacy, Zikei Hospital, Zikei Institute of Psychiatry, 100-2 Urayasu Honmachi, Minami-Ku, Okayama-shi, Okayama, 702-8508 Japan; 2 Scientific Research WorkS Peer Support Group (SRWS-PSG), Osaka, Japan; 3 Department of Pharmacy, Tokyo Medical University Hachioji Medical Center, Tatemachi, Hachioji, Tokyo, 1163, 193-0988 Japan

## Abstract

**Background:**

Topiramate has shown efficacy in reducing alcohol consumption and is increasingly used off-label for individuals with harmful alcohol use. However, findings regarding the moderating effect of the *GRIK1* rs2832407 single nucleotide polymorphism (SNP) on treatment outcomes remain inconsistent, highlighting the need for a review of the current evidence. We evaluated whether the *GRIK1* rs2832407 SNP moderates the efficacy and safety of topiramate treatment for alcohol use.

**Methods:**

We searched multiple databases including MEDLINE, Cochrane Library, ClinicalTrials.gov, and the International Clinical Trials Registry Platform up to December 1, 2024. Randomized controlled trials (RCTs) comparing treatment outcomes of topiramate in patients with alcohol use who were homozygous for the C allele at rs2832407 with those carrying one or more A alleles at rs2832407 were included. Primary outcomes were heavy drinking days (HDDs) and percentage of days abstinent (PDA), and the secondary outcome was side effects. Each outcome was evaluated using version 2 of the Cochrane Risk of Bias tool.

**Results:**

Our analysis included four RCTs. Among three studies evaluating HDDs, only one study demonstrated genotype effects, demonstrating a reduction in HDDs among CC carriers. Of two studies examining PDA, only one revealed genotype effects, indicating an increase in PDA. Side effects were evaluated in two studies, both of which assessed the severity of side effects, but with conflicting results regarding the effect of genotype.

**Conclusions:**

This systematic review highlights the current lack of sufficient evidence to confirm the pharmacogenetic effect of the *GRIK1* rs2832407 SNP on the efficacy or safety of topiramate treatment in individuals with harmful alcohol use.

**Trial registration:**

This research was prospectively registered with the Open Science Framework (https://osf.io/z2awu/).

**Supplementary Information:**

The online version contains supplementary material available at 10.1186/s40780-025-00449-y.

## Introduction

Alcohol is a well-known risk factor for early morbidity and mortality, accounting for 5.3% of all deaths worldwide [1]. The harmful use of alcohol leads to adverse social, occupational, and health consequences, while access to treatment remains very low, with less than 10% of affected individuals receiving medical care [2]. Pharmacotherapy, alongside psychosocial interventions, has become an essential component of treatment for harmful alcohol use [3, 4]. However, existing pharmacotherapies, including disulfiram, acamprosate, naltrexone, and nalmefene, show inconsistent effects across patients [5], leading to their underutilization. Therefore, developing new, more effective, and diverse pharmacological treatments for harmful alcohol use is essential to increase treatment uptake and improve patient outcomes.

Topiramate is an anticonvulsant with multiple neuropharmacological mechanisms, including modulation of GABAergic and glutamatergic neurotransmission [6–9]. Recently, topiramate has gained attention as a potential pharmacotherapy for alcohol use disorder (AUD) [10], showing efficacy comparable to that of naltrexone [11]. Various moderators of topiramate’s effects on drinking, both environmental and genetic, have been proposed. One hypothesized moderator is rs2832407, a single-nucleotide polymorphism (SNP) in the glutamate ionotropic receptor kainate-type subunit 1 (*GRIK1*) gene [12]. The glutamate receptors, particularly the kainate and α-amino-3-hydroxy-5-methyl-4-isoxazolepropionic acid subtypes, play a key role in regulating excitatory neurotransmission involved in alcohol-related behaviors, including alcohol consumption, reward processing, and withdrawal symptoms [13]. Given the critical role of glutamatergic pathways in the neurobiology of AUD, genetic variations in *GRIK1*, such as rs2832407, may influence individual differences in response to topiramate treatment.

Early pharmacogenetic studies have suggested that individuals homozygous for the C allele (CC) at rs2832407 exhibit greater reductions in heavy drinking days (HDDs) in response to topiramate treatment, and that this polymorphism was also associated with differences in topiramate-induced side effects and serum drug concentrations [12, 14–16]. However, subsequent genotype-stratified randomized controlled trials (RCTs) did not confirm an association between rs2832407 and variability in drinking outcomes following topiramate treatment [11, 17]. Given these mixed findings, a comprehensive evaluation of the genetic factors influencing topiramate response is warranted. Identifying specific alleles that modulate treatment efficacy could help refine pharmacogenetic approaches and personalize treatment strategies. Therefore, we conducted a systematic review to evaluate whether the *GRIK1* rs2832407 SNP influences the therapeutic effects of topiramate on alcohol consumption outcomes.

## Method

### Protocol and study design

This review adheres to the Preferred Reporting Items for Systematic Reviews and Meta-Analyses (PRISMA) 2020 statement for health-related research [18] (Supplemental Table 1). This research was prospectively registered with the Open Science Framework (https://osf.io/z2awu/). We searched for all types of RCTs, including crossover RCTs and cluster RCTs.

### Selection criteria

We included patients over 18 years old with problematic alcohol use (heavy drinking or AUD). AUD was diagnosed according to the Diagnostic and Statistical Manual of Mental Disorders (DSM)-IV, DSM-IV-Text Revision, DSM-5 or the International Classification of Diseases (ICD)-10. Some clinical trials enrolled participants based on heavy drinking criteria without requiring a formal diagnosis of AUD or explicit assessment of alcohol-related harm [12, 15]. Heavy drinking was defined based on alcohol consumption thresholds, whereas AUD was diagnosed according to behavioral, cognitive, and physiological criteria. We judged that including both patients with AUD and heavy drinkers without clinically verified harm would allow for a broader and more representative evaluation of treatment effects. All patients received treatment with topiramate. The exposure group consisted of patients with the CC genotype, while the control group included patients with either the AC or AA (AC/AA) genotype at rs2832407. This grouping was adopted because the majority of previous studies compared individuals with the CC genotype to those with AC/AA genotype [11, 12, 16, 19], rather than analyzing the three genotypes separately [15].

Concomitant psychosocial interventions were permitted, provided they were distributed equally across all study arms. Based on pre-defined eligibility criteria in the protocol, any pharmacotherapy combinations that contain topiramate were excluded because they do not allow for the evaluation of the effect of topiramate itself. A list of excluded reports is shown in Supplemental Table 2. All RCTs were deemed eligible for inclusion, regardless of publication status (e.g., published articles, unpublished manuscripts, conference abstracts, and letters), language, or country. If it was unclear whether a study met the review criteria, we contacted the original authors. If no response was received, we followed up with additional attempts to contact them.

### Search strategy

**Fig. 1:**
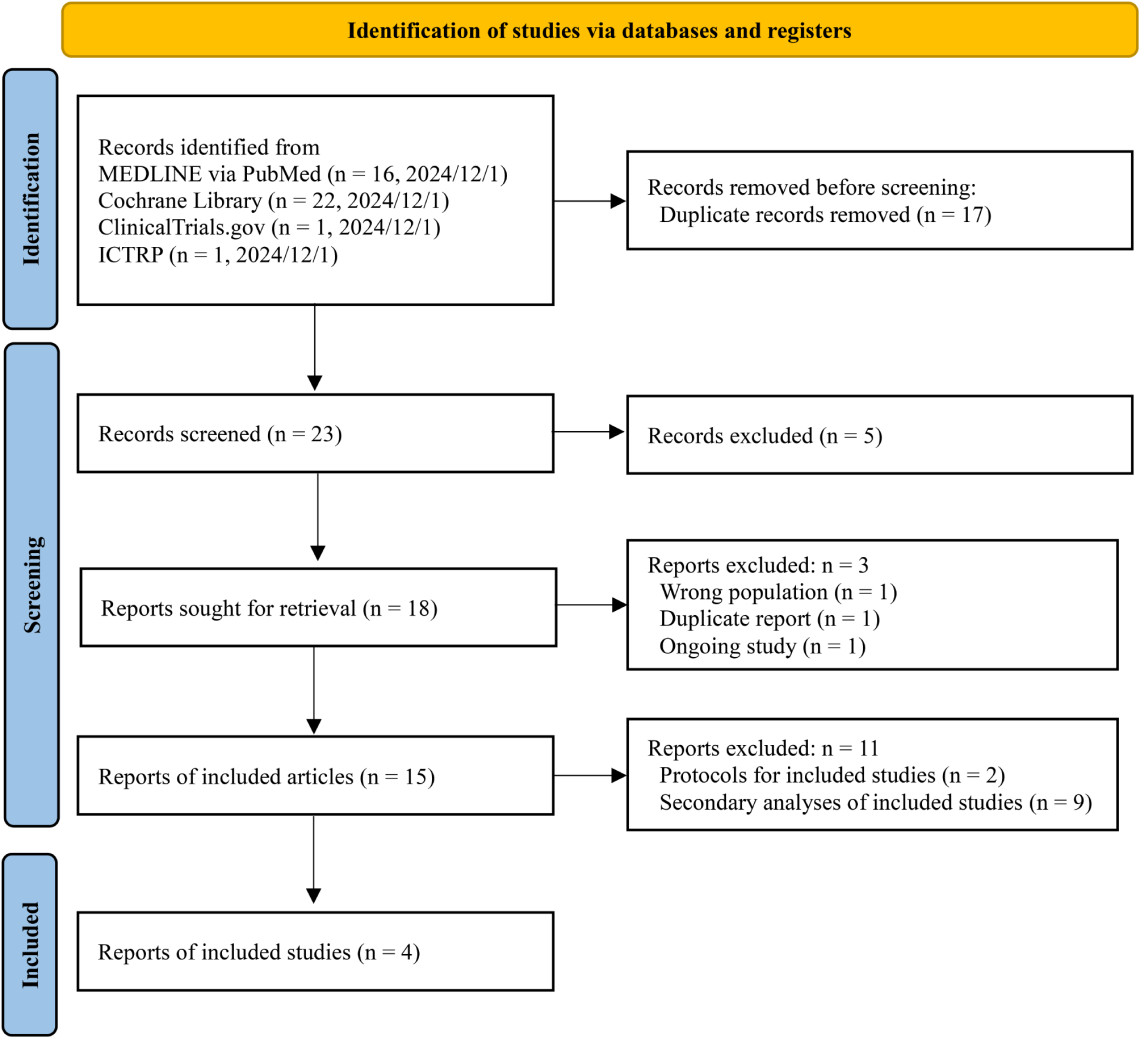
PRISMA flow diagram. CENTRAL, Cochrane Central Register of Controlled Trials; ICTRP, International Clinical Trials Registry Platform; MEDLINE, Medical Literature Analysis and Retrieval System Online; RCT, randomized controlled trial

We conducted a comprehensive search of the Cochrane Central Register of Controlled Trials via the Cochrane Library, MEDLINE via PubMed, ClinicalTrials.gov, and the International Clinical Trials Registry Platform up to December 1, 2024. Search terms including “alcoholism” and “alcohol drinking” were used to identify the target patient population, and the term “topiramate” were used to capture interventions. The *GRIK1* rs2832407 polymorphism, previously reported to be associated with topiramate response and alcohol use, was also included in the search strategy [12]. The detailed search strategy is shown in Supplemental Tables 3 and the PRISMA flow diagram in Fig. 1. Two authors (K.K. and S.M.) collected the data from studies included in the review.

### Outcome definitions and measures

The primary outcomes were HDDs and percentage of days abstinent (PDA) at the end of treatment, and the secondary outcome was side effects. These outcomes were defined according to the content described by individual studies. In addition, these outcomes were treated as continuous variables, we did not impute missing data [20].

### Data collection

We extracted study characteristics (first author, publication year), participant characteristics (age, sex, polymorphism in GRIK1), study design, drug intervention (the dosage and duration of topiramate administration), and outcome measures. We also recorded whether analyses of genotypic moderators were conducted prospectively (pre-specified at the time of randomization) or retrospectively (conducted post hoc based on genotype determined after randomization). This information was collected to assess the potential risk of bias arising from post hoc subgroup analyses by genotype, as retrospective genotyping may introduce bias that differs from the bias patterns observed in pre-specified stratified analyses. The extracted results were independently verified and modified by two reviewers (K.K., and S.M.). Any discrepancies in data reconciliation were resolved through discussions between the two authors until a consensus was reached.

### Risk of bias assessment

Two authors (K.K. and S.M.) independently assessed the potential risk of bias in each study using the Cochrane Risk of Bias tool designed for RCTs [21]. This tool evaluates five key domains: bias arising from the randomization process, bias due to deviations from the intended intervention, bias due to missing outcome data, bias in the measurement of the outcome, and bias in the selection of the reported result. Each study was assessed for potential sources of bias and categorized as “low risk,” “some concerns,” or “high risk.” Any discrepancies in the risk of bias assessments were resolved through discussions between the two authors (K.K. and S.M.) until consensus was achieved.

### Data synthesis and statistical analysis

We calculated the mean difference (MD) and 95% confidence intervals (CI) for the primary outcomes because both HDDs and PDA were assessed in terms of days of onset per week. The secondary outcome was expressed as the MD because all included studies assessed differences in topiramate genotypes and the severity of adverse effects. To evaluate the possibility of small study effects and their association with reporting bias, we planned to draw contour-enhanced funnel plots. All analyses were conducted using R version 4.2.3 (R Foundation for Statistical Computing, Vienna, Austria).

## Results

Overall, 16 records were identified from MEDLINE via PubMed, 22 records from the Cochrane Library, and one record each from ClinicalTrials.gov and the International Clinical Trials Registry Platform (Fig. 1). After screening, 15 reports were included, among which four RCTs were included in the analysis [11, 12, 15, 19]. The remaining 11 reports were from protocols or secondary analyses of the four included studies (Supplemental Table 4).

The summary of included studies is presented in Table 1. In total, three studies were performed in the United States of America [12, 15, 19], and 1 study were performed in Australia [11]. Two studies involved patients with heavy drinking [12, 15], and the other two studies involved patients with AUD [11, 19]. Analysis of genotypic modulators of treatment response was performed retrospectively in two trials [12, 15], and prospectively in the remaining two studies [11, 19]. The duration of treatment was 84 days in the three studies [11, 15, 19], the remaining study underwent a 32-day drug titration period, with the target dose maintained for up to 7 days and a 4-day stabilization period (Median 36 day) [12]. The mean age was 51.4 years with a standard deviation of 9.7 years. The overall proportion of men was 70.2%. Publication bias could not be assessed because fewer than ten studies reported both primary and secondary outcomes.

**Table 1:** Characteristics of the studies included in this review

References	Country	Patient Characteristics	Analyses of genotypic moderators	Study duration (days)	Dose of topiramate (mg)	Number of patients	Mean age (year)	SD of age (year)	Sex (Male) (%)
Ray LA 2009	USA	heavy drinking^a^	Retrospective	36 (Median)	200 or 300	CC^b^ 11	NI	NI	NI
						AC/AA^c^ 21	NI	NI	NI
Kranzler HR 2014	USA	heavy drinking^b^	Retrospective	84	200	CC^b^ 21	51.7	8.3	15 (71)
						AC^/^AA^c^ 35	50.1	6.8	22 (63)
Kranzler HR 2021	USA	AUD	Prospective	84	200	CC^b^ 30	53	11.9	22 (73)
						AC^/^AA^c^ 55	51.9	9.8	39 (71)
Morley KC 2024	Australia	AUD	Prospective	84	200	CC^b^ 28	NI	NI	NI
						AC^/^AA^c^ 44	NI	NI	NI

^a^An average weekly consumption of ≥ 18 standard drinks for men and ≥ 14 standard drinks for women

^a^An average weekly consumption of ≥ 24 standard drinks for men and ≥ 18 standard drinks for women

^b^CC, patients with homozygous for the C allele at rs2832407

^c^AC/AA, patients with one or more A alleles at rs2832407

AUD, Alcohol Use Disorder; NI, no information; SD, Standard Deviation; USA, United States of America

### Risk of bias within studies

**Fig. 2:**
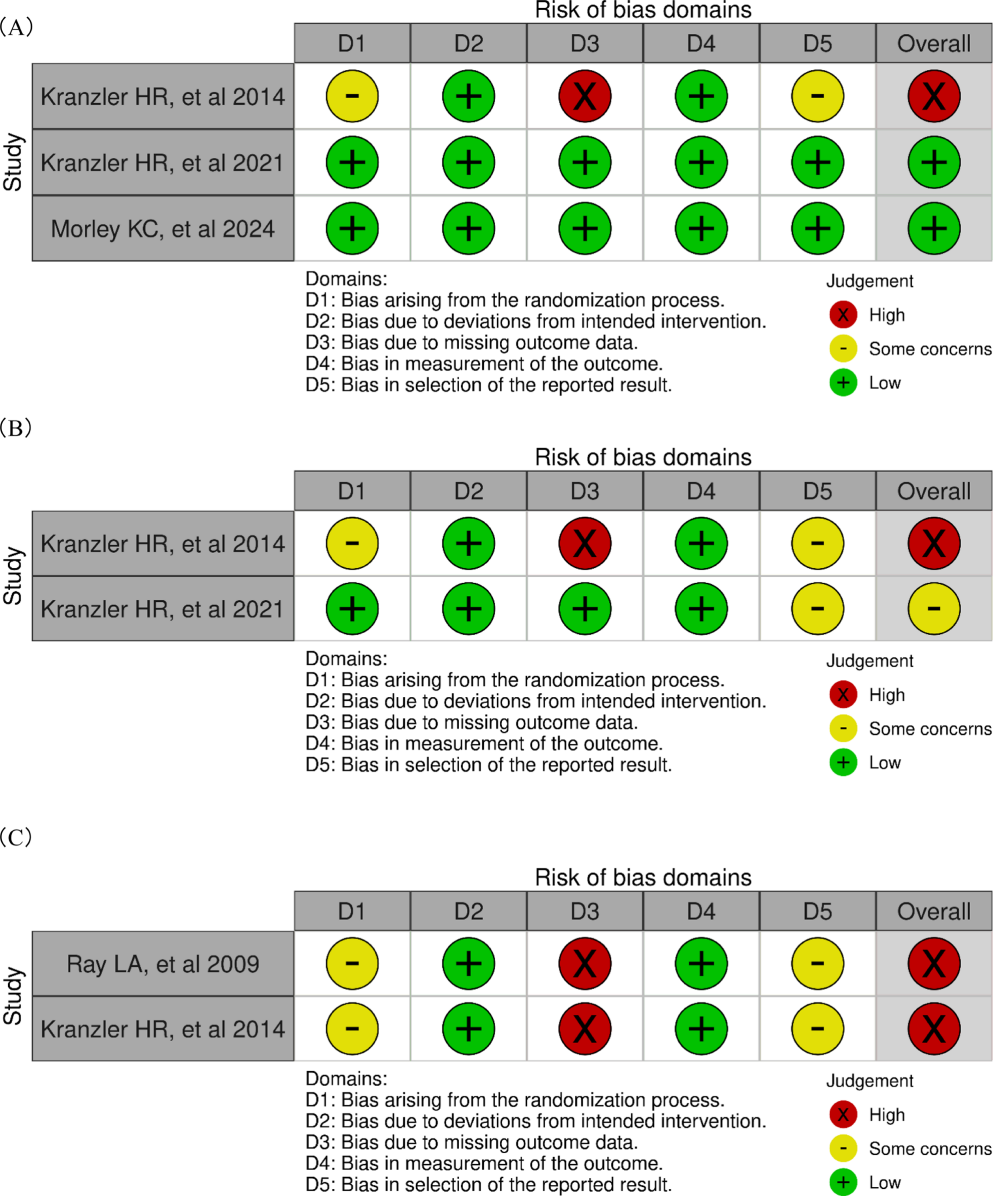
Risk of bias summary. (**A**) Heavy drinking days. (**B**) Percentage of days abstinent. (**C**) Severity of side effect. D1, bias arising from the randomization process; D2, bias due to deviations from intended interventions; D3, bias due to missing outcome data; D4, bias in measurement of the outcome; D5, bias in selection of the reported result. x, high risk of bias; −, some concerns; +, low risk of bias

As shown in Fig. 2A, the risk of bias for HDDs was rated as “high,” which also applied to PDA and severity of side effects (Fig. 2B and C). This is because dropout before or after topiramate administration prevented the assessment of all patients who received the treatment. Additionally, genotyping was conducted retrospectively in two included studies [12, 15]. Therefore, there is an additional concern for selection bias, as retrospective genotyping may have led to baseline imbalances between genetic subgroups that were not accounted for at the time of randomization. There is also a concern for reporting bias arising from post hoc subgroup analyses based on genotype.

### Primary outcomes

HDDs were reported in 3 studies [11, 15, 19], with the definition in all cases being ≥ 5 drinks/day for males and ≥ 4 drinks/day for females. In a study of patients with heavy drinking, the effect of topiramate was greater in those who were homozygous for the CC compared to those with AC/AA. (MD − 0.93, 95% CI − 1.27 to − 0.59) [15]. Meanwhile, in two studies involving patients with AUD, there was no significant difference in the response to topiramate between those who were homozygous for the CC and those with AC/AA. (MD − 0.08, 95% CI − 1.07 to 0.91) [19], (MD 0.96, 95% CI − 0.92 to 2.83) [11].

PDA was reported in two studies [15, 19]. Similarly, in a study of patients with heavy drinking, the effect of topiramate was greater in those who were homozygous for the CC compared to those with AC/AA (MD 0.66, 95% CI 0.29 to 1.03) [15], however, in a study of patients with AUD, there was no significant difference in the response to topiramate between those who were homozygous for the CC and those with AC/AA. (MD − 0.38, 95% CI − 1.48 to 0.72) [19].

### Secondary outcome

Side effects was reported in two studies [12, 15]. The two studies assessed the severity of adverse events using two measures: a continuous measure reflecting the mean number of 19 adverse events occurring at the target dosage and an adverse event severity score categorized as mild, moderate, or severe. One study demonstrated that the severity of side effects associated with topiramate use tended to be lower in those who were homozygous for the CC compared to those with AC/AA (MD − 2.50, 95% CI − 4.62 to − 0.38) [12]. In contrast, the other study found no association between genetic differences and severity of side effects (MD 0.79, 95% CI − 0.31 to 1.89) [15]. The remaining two studies were validated for side effects related to topiramate and comparator, but not for genotype-based effects [11, 19].

### Changes to the initial protocol and review

We initially planned to conduct a meta-analysis of the outcomes. However, the included studies exhibited considerable variability in patient backgrounds. Specifically, only two studies evaluated patients with AUD [11, 19], while the remaining two assessed patients with heavy drinking [12, 15]. In addition, the methods of genotyping differed. Consequently, the meta-analysis was discontinued. Similarly, the GRADE evaluation was also halted due to the inability to integrate the studies.

## Discussion

In this systematic review, we identified four RCTs that investigated the modulatory effect of the *GRIK1* rs2832407 polymorphism on treatment outcomes with topiramate in individuals with problematic alcohol use, including both heavy drinking and AUD. Due to substantial heterogeneity in patient backgrounds, definitions of drinking outcomes, and methods for genotyping, we determined that a meta-analysis was not appropriate. Instead, a qualitative synthesis of the findings was conducted.

Our systematic review highlights the current lack of sufficient evidence to support a pharmacogenetic effect of the rs2832407 SNP on topiramate treatment in individuals with alcohol use. This review underscores the need for further RCTs with larger sample sizes and standardized methodologies to advance precision medicine approaches for alcohol use.

The studies included in our review varied significantly in patient backgrounds and genetic analysis methods, posing challenges for interpretation. Specifically, we included patients with heavy drinking and those with AUD. These populations are often reported collectively [22, 23]; however, ‘patients with heavy drinking’ only indicates a high level of alcohol consumption and does not necessarily reflect awareness of their drinking problem, which varies among individuals. This individual difference may have influenced the discrepancy in results for the secondary outcome, severity of side effects. Specifically, severity of side effects was assessed in two studies that investigated individuals with heavy drinking [12, 15], but one study had a lower treatment completion rate than the other, suggesting that patients may be less motivated to seek treatment. These differences in motivation for alcohol treatment complicate interpretation. In addition, there were differences in how the moderators of rs2832407 were analyzed. Genotypes were retrospectively determined in two studies that reported that the SNP in rs2832407 affected the therapeutic effect of topiramate [12, 15]. In contrast, the genotype was determined prospectively in a study that found no association between differences in the SNP at rs2832407 and the therapeutic effect of topiramate [11, 19]. Previous reports have shown that differences in genotyping procedures are associated with increased risks of selection bias, population stratification, and genotyping errors, which may affect the reliability of estimated genetic effects [24]. Variability in genotyping methodology in our review may likewise have contributed to the observed heterogeneity in outcomes.

In addition to its relevance to topiramate treatment, *GRIK1* rs2832407 has also been reported to be associated with treatments other than topiramate for reducing alcohol use. In population pharmacokinetic models of topiramate in Chinese children with epilepsy, *GRIK1* rs2832407 was identified as a significant covariate decreasing clearance [25]. This pharmacogenetic approach is beneficial to selecting eligible patients because topiramate was reported to be poorly tolerated and to have almost twice the rate of adverse events that would induce discontinuation compared to placebo [26]. However, insufficient information exists to determine how the SNP in rs2832407 influences the efficacy of topiramate treatment, highlighting the need for further studies, including basic research [27].

Given the complexity of AUD treatment responses, it is essential to select treatments based on clinical and social factors relevant to individual patients. Many studies used pharmacogenetic approaches to investigate treatment responses in individuals with AUD [28–31]. While some research has examined pharmacogenetic aspects of AUD treatment medications, data supporting implementation in clinical practice remains limited [32]. Nevertheless, considering the restricted pharmacological options available for AUD, identifying patient subgroups likely to respond favorably is critical. Further research leveraging pharmacogenetic approaches is necessary to achieve precision medicine in AUD, enabling more effective treatments with fewer side effects for individual patients.

Our study has several limitations. First, the patients included in the study were heavy drinking and AUD, and their motivation for treatment differed. This complicates interpretation of the results, but we determined that a comprehensive evaluation was necessary because in real-world clinical practice, this difference in motivation varies among individual patients. Second, the methods used to investigate genetic polymorphisms differed across studies, with genotype effects appearing stronger in those that conducted retrospective genotyping than in those that used prospective approaches. This heterogeneity in genotyping methodology may have contributed not only to selection bias but also to publication bias, as studies showing stronger genotype effects might have been more likely to be published. Finally, some studies could not collect background information on patients with rs2832407 homozygous CC versus those with other alleles. Despite these limitations, this is the first systematic review of the modulatory effects of the rs2832407 SNP on topiramate response. Although no evidence was found supporting the pharmacogenetic effect of the rs2832407 SNP on the response to topiramate treatment for alcohol use, investigating SNPs associated with topiramate’s moderating effects using a pharmacogenetic approach is clinically important for advancing personalized medicine in individuals with harmful alcohol use.

## Conclusions

This systematic review highlights the current lack of sufficient evidence to support the pharmacogenetic effect of the rs2832407 SNP on the response to topiramate treatment in individuals with harmful alcohol use. Further RCTs are desired to obtain evidence for personalizing topiramate treatment in individuals with harmful alcohol use.

## Electronic supplementary material

Below is the link to the electronic supplementary material.

Supplementary Material 1

## Data Availability

Data supporting the findings of this study are available at Open Science Framework (https://osf.io/z2awu/). Further data are available from the corresponding author upon reasonable request (kottanketty@gmail.com).

## Abbreviations

AUD: Alcohol use disorder
CI: Confidence intervals
DSM: Diagnostic and statistical manual of mental disorders
GRIK1: Glutamate ionotropic receptor kainate-type subunit 1
HDDs: Heavy drinking days
ICD: International classification of diseases
MD: Mean difference
PDA: Percentage of days abstinent
PRISMA: Preferred reporting items for systematic reviews and meta-analyses
RCTs: Randomized controlled trials
