# Supplementary material for: GRIK1 genotype and effect of topiramate for alcohol use: a systematic review
Source: J Pharm Health Care Sci. 2025 May 20;11:42. doi: 10.1186/s40780-025-00449-y (PMC12093777; doi:10.1186/s40780-025-00449-y)
Supplement: Supplementary file 1 — Supplementary Material 1 [file 40780_2025_449_MOESM1_ESM.docx]

Supporting Information

**GRIK1 genotype and effect of topiramate on alcohol drinking: a systematic review**

**Contents**

Supplemental Table 1. PRISMA checklist.

Supplemental Table 2. List of excluded studies.

Supplemental Table 3 Search strategy.

Supplemental Table 4 List of protocol or secondary analysis studies.

**Supplemental Table 1 PRISMA checklist**

| **Section and Topic** | **Item #** | **Checklist item** | **Location where item is reported** | |
| --- | --- | --- | --- | --- |
| **TITLE** | | |  | |
| Title | 1 | Identify the report as a systematic review. | 1 | |
| **ABSTRACT** | | |  | |
| Abstract | 5,6 | See the PRISMA 2020 for Abstracts checklist. | 2,3 | |
| **INTRODUCTION** | | |  | |
| Rationale | 7 | Describe the rationale for the review in the context of existing knowledge. | 4,5 | |
| Objectives | 7,8 | Provide an explicit statement of the objective(s) or question(s) the review addresses. | 4,5 | |
| **METHODS** | | |  | |
| Eligibility criteria | 8,9 | Specify the inclusion and exclusion criteria for the review and how studies were grouped for the syntheses. | 4-6 | |
| Information sources | 9,10 | Specify all databases, registers, websites, organisations, reference lists and other sources searched or consulted to identify studies. Specify the date when each source was last searched or consulted. | 4-6 |  |
| Search strategy | 9,10 | Present the full search strategies for all databases, registers and websites, including any filters and limits used. | 7 | |
| Selection process | 9,10 | Specify the methods used to decide whether a study met the inclusion criteria of the review, including how many reviewers screened each record and each report retrieved, whether they worked independently, and if applicable, details of automation tools used in the process. | 7 | |
| Data collection process | 10,11 | Specify the methods used to collect data from reports, including how many reviewers collected data from each report, whether they worked independently, any processes for obtaining or confirming data from study investigators, and if applicable, details of automation tools used in the process. | 7 | |
| Data items | 10,11 | List and define all outcomes for which data were sought. Specify whether all results that were compatible with each outcome domain in each study were sought (e.g. for all measures, time points, analyses), and if not, the methods used to decide which results to collect. | 7 | |
|  | 10,11 | List and define all other variables for which data were sought (e.g. participant and intervention characteristics, funding sources). Describe any assumptions made about any missing or unclear information. | 7 | |
| Study risk of bias assessment | 11,12 | Specify the methods used to assess risk of bias in the included studies, including details of the tool(s) used, how many reviewers assessed each study and whether they worked independently, and if applicable, details of automation tools used in the process. | 8 | |
| Effect measures | 10 | Specify for each outcome the effect measure(s) (e.g. risk ratio, mean difference) used in the synthesis or presentation of results. | 8 | |
| Synthesis methods | 11,12 | Describe the processes used to decide which studies were eligible for each synthesis (e.g. tabulating the study intervention characteristics and comparing against the planned groups for each synthesis (item #5)). | 8 | |
|  | 11,12 | Describe any methods required to prepare the data for presentation or synthesis, such as handling of missing summary statistics, or data conversions. | 8 | |
|  | 11,12 | Describe any methods used to tabulate or visually display results of individual studies and syntheses. | 8 | |
|  | 11,12 | Describe any methods used to synthesize results and provide a rationale for the choice(s). If meta-analysis was performed, describe the model(s), method(s) to identify the presence and extent of statistical heterogeneity, and software package(s) used. | 8 | |
|  | 11,12 | Describe any methods used to explore possible causes of heterogeneity among study results (e.g. subgroup analysis, meta-regression). | 8 | |
|  | 11,12 | Describe any sensitivity analyses conducted to assess robustness of the synthesized results. | 8 | |
| Reporting bias assessment | 11,12 | Describe any methods used to assess risk of bias due to missing results in a synthesis (arising from reporting biases). | 8 | |
| Certainty assessment | 12 | Describe any methods used to assess certainty (or confidence) in the body of evidence for an outcome. | N/A | |
| **RESULTS** | | |  | |
| Study selection | 14 | Describe the results of the search and selection process, from the number of records identified in the search to the number of studies included in the review, ideally using a flow diagram. | 9 | |
|  | 14 | Cite studies that might appear to meet the inclusion criteria, but which were excluded, and explain why they were excluded. | 9 | |
| Study characteristics | 14 | Cite each included study and present its characteristics. | 9 | |
| Risk of bias in studies | 15 | Present assessments of risk of bias for each included study. | 10 | |
| Results of individual studies | 15-20 | For all outcomes, present, for each study: (a) summary statistics for each group (where appropriate) and (b) an effect estimate and its precision (e.g. confidence/credible interval), ideally using structured tables or plots. | 10,11 | |
| Results of syntheses | 15-20 | For each synthesis, briefly summarise the characteristics and risk of bias among contributing studies. | 10,11 | |
|  | 15-20 | Present results of all statistical syntheses conducted. If meta-analysis was done, present for each the summary estimate and its precision (e.g. confidence/credible interval) and measures of statistical heterogeneity. If comparing groups, describe the direction of the effect. | N/A | |
|  | 15-20 | Present results of all investigations of possible causes of heterogeneity among study results. | N/A | |
|  | 15-20 | Present results of all sensitivity analyses conducted to assess the robustness of the synthesized results. | N/A | |
| Reporting biases | 15-20 | Present assessments of risk of bias due to missing results (arising from reporting biases) for each synthesis assessed. | 10 | |
| Certainty of evidence | 15-20 | Present assessments of certainty (or confidence) in the body of evidence for each outcome assessed. | N/A | |
| **DISCUSSION** | | |  | |
| Discussion | 20-22 | Provide a general interpretation of the results in the context of other evidence. | 11-14 | |
|  | 22,23 | Discuss any limitations of the evidence included in the review. | 11-14 | |
|  | 22,23 | Discuss any limitations of the review processes used. | 11-14 | |
|  | 23,24 | Discuss implications of the results for practice, policy, and future research. | 11-14 | |
| **OTHER INFORMATION** | | |  | |
| Registration and protocol | 8 | Provide registration information for the review, including register name and registration number, or state that the review was not registered. | 5 | |
|  | 8 | Indicate where the review protocol can be accessed, or state that a protocol was not prepared. | 5 | |
|  | 13 | Describe and explain any amendments to information provided at registration or in the protocol. | 5 | |
| Support | 4 | Describe sources of financial or non-financial support for the review, and the role of the funders or sponsors in the review. | Title | |
| Competing interests | 4 | Declare any competing interests of review authors. | Title | |
| Availability of data, code and other materials | 4 | Report which of the following are publicly available and where they can be found: template data collection forms; data extracted from included studies; data used for all analyses; analytic code; any other materials used in the review. | Title | |

**Supplemental Table 2. List of excluded studies.**

| **Number** | **Reason for exclusion** | **References** |
| --- | --- | --- |
| 1 | Wrong population | Arias AJ, Feinn R, Kranzler HR, et al. Allelic association of the GluR5 kainate receptor subunit gene GRIK1 with adverse effects of topiramate. Neuropsychopharmacology, 2010, 35, S114‐S115. https://doi.org/10.1038/npp.2010.216 |
| 2 | Duplicate report | Morley KC, Kranzler HR, Luquin N, et al. Topiramate versus naltrexone for alcohol use disorder: A genotype-stratified double-blind randomized controlled trial. Am J Psychiatry. 2024, 181(5), 403-411. https://doi.org/10.1176/appi.ajp.20230666 |
| 3 | Ongoing study | NCT03667846. Leveraging Biomarkers for Personalized Treatment of Alcohol Use Disorder Comorbid With PTSD. https://clinicaltrials.gov/study/NCT03667846 |

**Supplemental Table 3. Search strategy**

(A) MEDLINE (via PubMed) (Performed on December 1, 2024)

#1 Alcoholism [mh] or Alcohol Drinking [mh]

#2 alcohol [tiab] or drink* [tiab]

#3 addict* [tiab] or abus* [tiab] or dependen* [tiab] or disorder* [tiab] or consumption* [tiab]

#4 #2 and #3

#5 #1 or #4

#6 Topiramate [mh]

#7 Topiramate [tiab]

#8 #6 or #7

#9 rs2832407 [tiab]

#10 randomized controlled trial [pt]
#11 controlled clinical trial [pt]
#12 randomized [tiab]
#13 placebo [tiab]
#14 drug therapy [sh]
#15 randomly [tiab]
#16 trial [tiab]
#17 groups [tiab]
#18 #10 or #11 or #12 or #13 or #14 or #15 or #16 or #17
#19 animals [mh] not humans [mh]
#20 #18 not #19

#24 #5 and #8 and #9 and #20

(B) Cochrane Library (Performed on December 1, 2024)

#1. MeSH descriptor: [Alcohols] in all MeSH products

#2. ((alcohol*) near (abus* or addict* or dependen* or disorder* or drink* or consumption*))

#3. MeSH descriptor: [Alcohol Drinking] explode all trees

#4. #1 or #2 or #3

#5. (Topiramate)

#6. rs2832407

#7. #5 and #6

#8. #4 and #7

(C) ClinicalTrials.gov (Performed on December 1, 2024)

Advanced search

Condition or disease: Alcoholism or Alcohol Drinking

Intervention: Topiramate

Study Type: Interventional Studies (Clinical Trials)

Study Results: All Studies

(D) International Clinical Trials Registry Platform (Performed on December 1, 2024)

Advanced search

Condition or disease: Alcoholism or Alcohol Drinking

Intervention: Topiramate

Recruitment status is ALL

**Supplemental Table 4 List of protocol or secondary analysis studies**

| **Number** | **Main article** | **Protocol References** |
| --- | --- | --- |
| 1 | ・Morley KC, Kranzler HR, Luquin N, et al. Topiramate versus naltrexone for alcohol use disorder: A genotype-stratified double-blind randomized controlled trial. Am J Psychiatry. 2024, 181(5), 403-411. https://doi.org/10.1176/appi.ajp.20230666 | ・Morley KC, Kranzler HR, Luquin N, et al. Topiramate versus naltrexone for alcohol use disorder: study protocol for a genotype-stratified, double-blind randomised controlled trial (TOP study). Trials. 2018, 19(1), 443. https://doi.org/10.1186/s13063-018-2824-z |
| 2 | ・Morley KC, Kranzler HR, Luquin N, et al. Topiramate versus naltrexone for alcohol use disorder: A genotype-stratified double-blind randomized controlled trial. Am J Psychiatry. 2024, 181(5), 403-411. https://doi.org/10.1176/appi.ajp.20230666 | ・NCT03479086. Defining the Clinical Role of Topiramate in the Treatment of Alcohol Dependence in Australia. https://clinicaltrials.gov/study/NCT03479086 |
| **Number** | **Main article** | **References for Secondary Analyses** |
| 1 | ・Kranzler HR, Morris PE, Pond T, et al. Prospective randomized pharmacogenetic study of topiramate for treating alcohol use disorder. *Neuropsychopharmacology*. 2021, 46(8), 1407-1413. https://doi.org/10.1038/s41386-021-01013-6 | ・Votaw VR, Witkiewitz K, Van Horn ML, Crist RC, Pond T, Kranzler HR. An intensive longitudinal examination of topiramate treatment for alcohol use disorder: a secondary analysis of data from a randomized controlled trial. *Addiction*. 2023;118(6):1040-1052. |
| 2 | ・Kranzler HR, Morris PE, Pond T, et al. Prospective randomized pharmacogenetic study of topiramate for treating alcohol use disorder. *Neuropsychopharmacology*. 2021, 46(8), 1407-1413. <https://doi.org/10.1038/s41386-021-01013-6>  ・Kranzler HR, Covault J, Feinn R, et al. Topiramate treatment for heavy drinkers: moderation by a GRIK1 polymorphism. *Am J Psychiatry*. 2014, 171(4), 445-452. https://doi.org/10.1176/appi.ajp.2013.13081014 | ・Kranzler HR, Feinn R, Pond T, et al. Post-treatment effects of topiramate on alcohol-related outcomes: A combined analysis of two placebo-controlled trials. Addict Biol. 2022, 27(2), e13130. https://doi.org/10.1111/adb.13130 |
| 3 | ・Kranzler HR, Morris PE, Pond T, et al. Prospective randomized pharmacogenetic study of topiramate for treating alcohol use disorder. *Neuropsychopharmacology*. 2021, 46(8), 1407-1413. <https://doi.org/10.1038/s41386-021-01013-6>  ・Kranzler HR, Covault J, Feinn R, et al. Topiramate treatment for heavy drinkers: moderation by a GRIK1 polymorphism. *Am J Psychiatry*. 2014, 171(4), 445-452. <https://doi.org/10.1176/appi.ajp.2013.13081014> | ・Kranzler HR, Hartwell EE, Feinn R, et al. Combined analysis of the moderating effect of a GRIK1 polymorphism on the effects of topiramate for treating alcohol use disorder. Drug Alcohol Depend. 2021, 225(108762), 108762. https://doi.org/10.1016/j.drugalcdep.2021.108762 |
| 4 | ・Kranzler HR, Covault J, Feinn R, et al. Topiramate treatment for heavy drinkers: moderation by a GRIK1 polymorphism. *Am J Psychiatry*. 2014, 171(4), 445-452. https://doi.org/10.1176/appi.ajp.2013.13081014 | ・Feinn R, Curtis B, Kranzler HR. Balancing risk and benefit in heavy drinkers treated with topiramate: implications for personalized care: Implications for personalized care. J Clin Psychiatry. 2016, 77(3), e278-82. https://doi.org/10.4088/JCP.15m10053 |
| 5 | ・Kranzler HR, Covault J, Feinn R, et al. Topiramate treatment for heavy drinkers: moderation by a GRIK1 polymorphism. *Am J Psychiatry*. 2014, 171(4), 445-452. https://doi.org/10.1176/appi.ajp.2013.13081014 | ・Kranzler HR, Wetherill R, Feinn R, Pond T, Gelernter J, Covault J. Post-treatment effects of topiramate treatment for heavy drinking. *Alcohol Clin Exp Res*. 2014, 38(12), 3017-3023. https://doi.org/10.1111/acer.12578 |
| 6 | ・Kranzler HR, Covault J, Feinn R, et al. Topiramate treatment for heavy drinkers: moderation by a GRIK1 polymorphism. *Am J Psychiatry*. 2014, 171(4), 445-452. https://doi.org/10.1176/appi.ajp.2013.13081014 | ・Kranzler HR, Armeli S, Wetherill R, et al. Self-efficacy mediates the effects of topiramate and GRIK1 genotype on drinking: Topiramate and self-efficacy. Addict Biol. 2016, 21(2), 450-459. https://doi.org/10.1111/adb.12207 |

| 7 | ・Kranzler HR, Covault J, Feinn R, et al. Topiramate treatment for heavy drinkers: moderation by a GRIK1 polymorphism. *Am J Psychiatry*. 2014, 171(4), 445-452. https://doi.org/10.1176/appi.ajp.2013.13081014 | ・Kranzler HR, Armeli S, Tennen H, Gelernter J, Covault J. GRIK1 genotype and daily expectations of alcohol’s positive effects moderate the reduction of heavy drinking by topiramate. Exp Clin Psychopharmacol. 2014, 22(6), 494-501. https://doi.org/10.1037/a0038350 |
| --- | --- | --- |
| 8 | ・Kranzler HR, Covault J, Feinn R, et al. Topiramate treatment for heavy drinkers: moderation by a GRIK1 polymorphism. *Am J Psychiatry*. 2014, 171(4), 445-452. https://doi.org/10.1176/appi.ajp.2013.13081014 | ・Kranzler HR, Feinn R, Gelernter J, Pond T, Covault J. Topiramate’s reduction of body mass index in heavy drinkers: lack of moderation by a GRIK1 polymorphism. Exp Clin Psychopharmacol. 2014, 22(5), 419-423. https://doi.org/10.1037/a0037309 |
| 9 | ・Kranzler HR, Covault J, Feinn R, et al. Topiramate treatment for heavy drinkers: moderation by a GRIK1 polymorphism. *Am J Psychiatry*. 2014, 171(4), 445-452. https://doi.org/10.1176/appi.ajp.2013.13081014 | ・Kranzler HR, Armeli S, Feinn R, Tennen H, Gelernter J, Covault J. GRIK1 genotype moderates topiramate’s effects on daily drinking level, expectations of alcohol’s positive effects and desire to drink. Int J Neuropsychopharmacol. 2014, 17(10), 1549-1556. https://doi.org/10.1017/S1461145714000510 |
